# Supplementary material for: Automated long axial field of view PET image processing and kinetic modelling with the TurBO toolbox
Source: Eur J Nucl Med Mol Imaging. 2026 Jan 31;53(6):4162–74. doi: 10.1007/s00259-026-07769-7 (PMC13121325; doi:10.1007/s00259-026-07769-7)
Supplement: Supplementary file 1 — Supplementary file1 (PDF 2478 KB) [file 259_2026_7769_MOESM1_ESM.pdf]

Automated Long Axial Field of View PET Image Processing and Kinetic Modelling with the TurBO Toolbox

Jouni Tuisku*[1,2], Santeri Palonen*[1,2], Henri Kärpijoki[1,2], Aino Latva-Rasku[1,2,3], Nelli Tuomola[1,2,3], Harri Harju[1,2], Sergey V. Nesterov[1,2], Vesa Oikonen[1,2], Hidehiro Iida[1,2], Jarmo Teuho[1,2], Chunlei Han[1,2], Tomi Karjalainen[1,2], Anna K. Kirjavainen[1,5], Johan Rajader[1,4], Riku Klén[1,2], Pirjo Nuutila[1,2,3], Juhani Knuuti[1,2], Lauri Nummenmaa[1,2,5]

[1]Turku PET Centre, University of Turku, Turku, Finland
[2]Turku PET Centre, Turku University Hospital, Turku, Finland
[3]Department of Endocrinology, Turku University Hospital, Turku, Finland
[4]Turku PET Centre, Accelerator Laboratory, Åbo Akademi University, Turku, Finland
[5]Turku PET Centre, Radiopharmaceutical Chemistry Laboratory, University of Turku, Turku, Finland.
[6]Department of Psychology, University of Turku, Turku, Finland

*Contributed equally to this study

SUPPLEMENTARY INFORMATION

MATERIALS AND METHODS

[^{15}O]H_2O model equations

One-tissue compartmental model is described using the following differential equation:

$$\frac{dC_T(t)}{dt} = K_1 C_A(t) - k_2 C_T(t) \tag{1}$$

Assuming that initial concentration in tissue compartment is zero ($C_T(0) = 0$),
eq (1) can be integrated to provide the tissue concentration at time T:

$$C_T(T) = K_1 \int_0^T C_A(t)dt - k_2 \int_0^T C_T(t)dt \tag{2}$$

The measured PET radioactivity concentration in tissue is contaminated by spillover from adjacent blood vessels C_B and vascular volume V_B inside the measured region $C_{PET}(t)$, which can be formulated as follows:

$$C_{PET}(t) = V_B C_B(t) + C_T(t) \tag{3}$$

Substituting $C_T(t)$ in eq (3) with eq (2) gives the following equation:

$$C_{PET}(t) = V_B C_B(t) + K_1 \int_0^T C_A(t)dt - k_2 \int_0^T C_T(t)dt \tag{4}$$

Integration and rearrangement of eq (3) gives

$$\int_0^T C_T(t)dt = \int_0^T C_{PET}(t)dt - V_B \int_0^T C_B(t)dt \tag{5}$$

which is then substituted in eq (4), providing the following equation:

$$C_{PET}(t) = V_B C_B(t) + K_1 \int_0^T C_A(t)dt - k_2 \int_0^T C_{PET}(t)dt + k_2 V_B \int_0^T C_B(t)dt \tag{6}$$

If the radioactivity concentration in blood can be represented by the model input function, as is the case with $[^{15}O]H_2O$, then $C_B(t) = C_A(t)$ and $V_B = V_A$, and then eq (6) simplifies to eq (7):

$$C_{PET}(t) = V_A C_A(t) + (K_1 + V_A k_2) \int_0^T C_A(t)dt - k_2 \int_0^T C_{PET}(t)dt \tag{7}$$

Equation (7) is a multilinear equation $y = p_1\, x_1 + p_2 x_2 + p_3 x_3$, where the coefficients

$$V_A = p_1$$
$$k_2 = p_3$$
$$K_1 = p_2 - V_A k_2$$

can be estimated with least squares method [1]. Particularly, non-negative least squares (NNLS) [2] is well-suited method for estimating the coefficients.

The differential equation (1) can also be directly solved using convolution:

$$C_T(t) = K_1 C_A * e^{-k_2 t} \tag{8}$$

where $C_T(t)$ is the radioactivity concentration in tissue, $C_A(t)$ is the arterial input function and * is the convolution operator. By denoting $C_B(t) = C_A(t)$ and $V_B = V_A$, and substituting eq (8) to eq (3), we get

$$C_{PET}(t) = K_1 C_A * e^{-k_2 t} + V_A C_A(t) \tag{9}$$

The equation (9) can be rewritten as a multilinear equation [3]:

$$C_T(t) = \theta_1 B(k_2, t) + \theta_2 C_A(t),$$

using basis functions

$$B(k_2, t) = C_A(t) * e^{-k_2 t},$$

and where the coefficients

$$\theta_1 = K_1$$
$$\theta_2 = V_A$$

can be estimated with NNLS.

$[^{15}O]H_2O$ liver modelling

The liver is supplied by the hepatic artery and the portal vein. The hepatic artery delivers tracer concentrations matching other arteries, while the portal vein provides a delayed, dispersed input due to passage through gut compartment [3].

The concentration of radiowater in the liver was modelled using the one-tissue compartmental model with the two input functions C_A and C_{PV} [3,4]:

$$\frac{dC_T(t)}{dt} = f_A C_A(t) + f_{PV} C_{PV}(t) - \frac{f_A + f_{PV}}{p} C_T(t) \quad (10)$$

where $p=K_1/k_2$ is the partition coefficient of water, C_{PET} is the measured PET activity concentration, C_T is the tissue activity concentration, C_A is the arterial activity concentration corrected for radiotracer delay, C_{PV} is the activity concentration of portal vein corrected for radiotracer delay, f_A and f_{PV} represent arterial and portal vein blood flow, and V_A is the arterial volume fraction.

Similarly to the aortic input, the portal vein input function C_{PV} can in principle be derived from the LAFOV PET image. However, the small size of the CT-segmented ROI can limit the reliability of the resulting image-derived portal vein input. Therefore, C_{PV} was simulated from aorta IDIF using the equation

$$C_{PV} = k_{GI} C_A \otimes \mathrm{e}^{-\mathrm{k}_{\mathrm{GI}} t} \quad (11)$$

where k_{GI} represents the diffusion rate in the gut system [3].

To estimate the liver parameters using the standard one-tissue compartment model, a combined input function was constructed as follows. First, the full dual-input model was fitted to the liver time–activity curve using NNLS to estimate the model parameters f_A, f_{PV}, k_2, V_A and k_{GI}, including the delay parameters for arterial and portal vein curves. The estimated parameters together with the simulated a portal vein input curve C_{PV} were then used to create a combined input curve:

$$C_{liverIF}(t) = \frac{f_A C_{IDIF}(t) + f_{PV} C_{PV}(t)}{f_A + f_{PV}}. \quad (12)$$

Data acquisition

The subject's demographic details are listed in **Table S1**.

Table S1. Study sample characteristics.

Demographic	[^{15}O]H_2O	[^{18}F]FDG
n (Males/Females)	8 / 13	4 / 12
Age y (mean ± sd)	65.4 ± 8.9	38.0 ± 7.0
Dose MBq (mean ± sd)	359.6 ± 23.8	171.8 ± 8.8
BMI kg/m2 (mean ± sd)	28.0 ± 4.9	30.6 ± 9.2

[^{15}O]H_2O PET data were acquired for 280 s following 359.6 ± 23.8 MBq bolus injection over 10–15 seconds (Radiowater Generator, Hidex Oy, Finland). Imaging started 30 seconds after

the start of the bolus injection. The data were reconstructed into 24 frames (14 × 5 s, 3 × 10 s, 3 × 20 s, 4 × 30 s) using an image matrix of 220 × 220 × 380 and a voxel size of 1.65 × 1.65 × 2.80 mm³. Reconstruction was performed with an ordered-subsets expectation maximization (OSEM) algorithm (3 iterations, 5 subsets) using point-spread function and time-of-flight modelling, and included corrections for decay, randoms, attenuation, and scatter.

[^{18}F]FDG PET data were acquired for 55 minutes during hyperinsulinemic, euglycemic clamp after 171.8 ± 8.8 MBq bolus injection. Before the scan, two venous catheters were inserted in the opposite forearms - one for the insulin and glucose infusions and for injecting [^{18}F]FDG, and the other for collecting venous blood samples, arterialised by placing a hot water bottle distally on the arm. After the collection of fasting plasma blood samples, hyperinsulinemic, euglycemic clamp was started [5]. Insulin (Actrapid, Novo Nordisk A/S, Bagsvaerd, Denmark) was administered with a dose of 40 mU/min/m^2 of body surface area, and a variable rate of 20% glucose was infused based on plasma glucose measurements performed every 5–10 min to maintain euglycemia (plasma glucose 5.0 mmol/L). The [^{18}F]FDG PET scan was started 60 minutes after the start of insulin infusion under steady euglycemia. Data were reconstructed using OSEM algorithm (4 iterations, 5 subsets) using point-spread function and time-of-flight modelling, and included corrections for decay, randoms, attenuation, and scatter. The data were reconstructed into 34 frames (l2 x 5s, 6 x10s, 6 x 20s, 2 x 60s, 2 x 120s, 4 x 300s, 2x 600s) with 440 x 440 x 354 matrix size and 1.65 x 1.65 x 3.0 mm^3 voxel-size.

Prior to the PET image acquisition, low-dose total-body CT images (effective dose 1.2 mSv) were acquired and reconstructed to 512 × 512 × 380 image matrix with a voxel size of 0.977 × 0.977 × 2.80 mm^3.

Quality control plots for representative [^{15}O]H_2O subject:

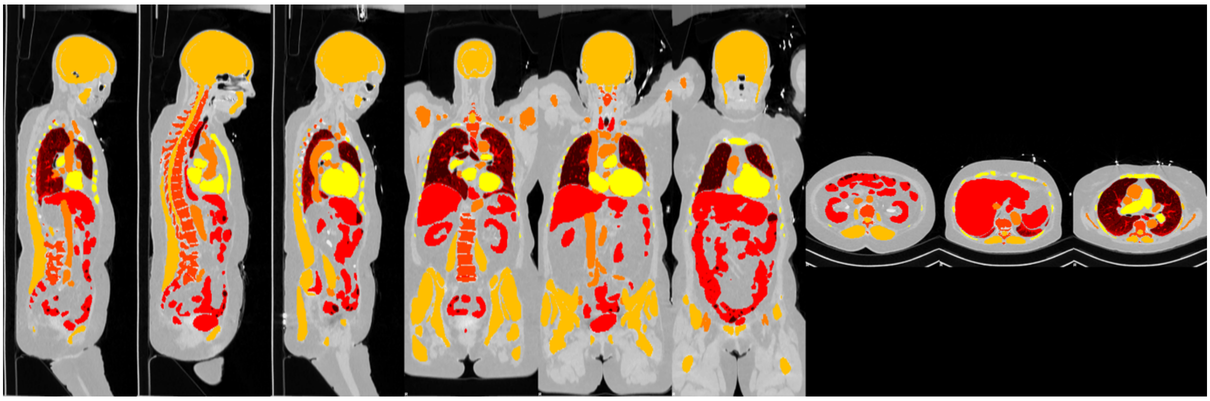

Figure S1. CT segments overlaid on CT image.

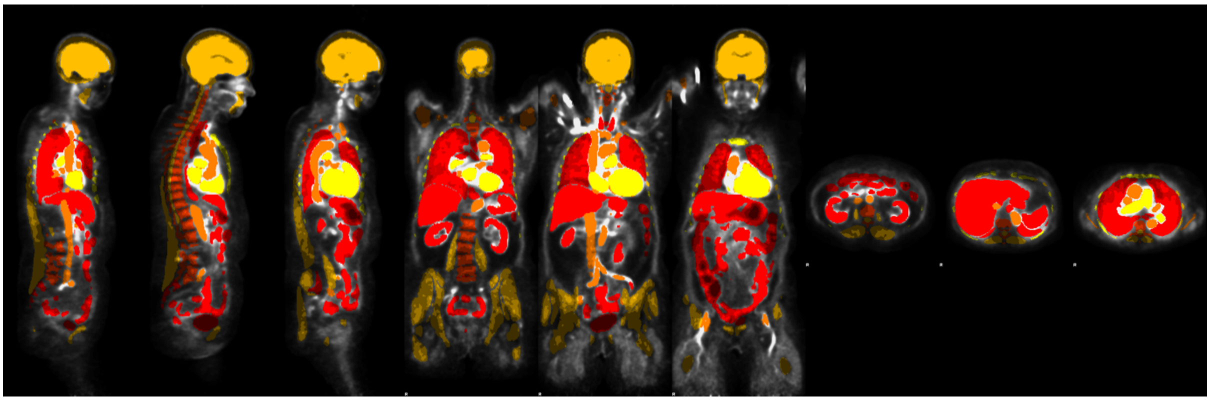

Figure S2. CT segments overlaid on mean PET image.

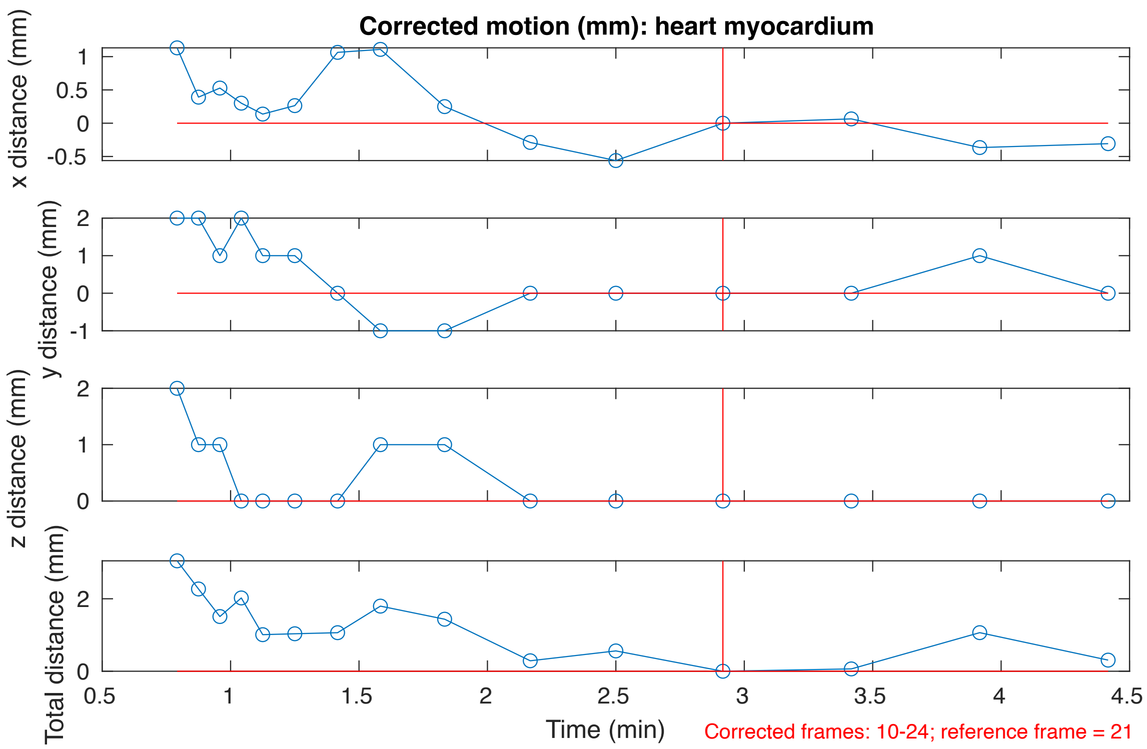

Figure S3. Corrected motion for heart myocardium

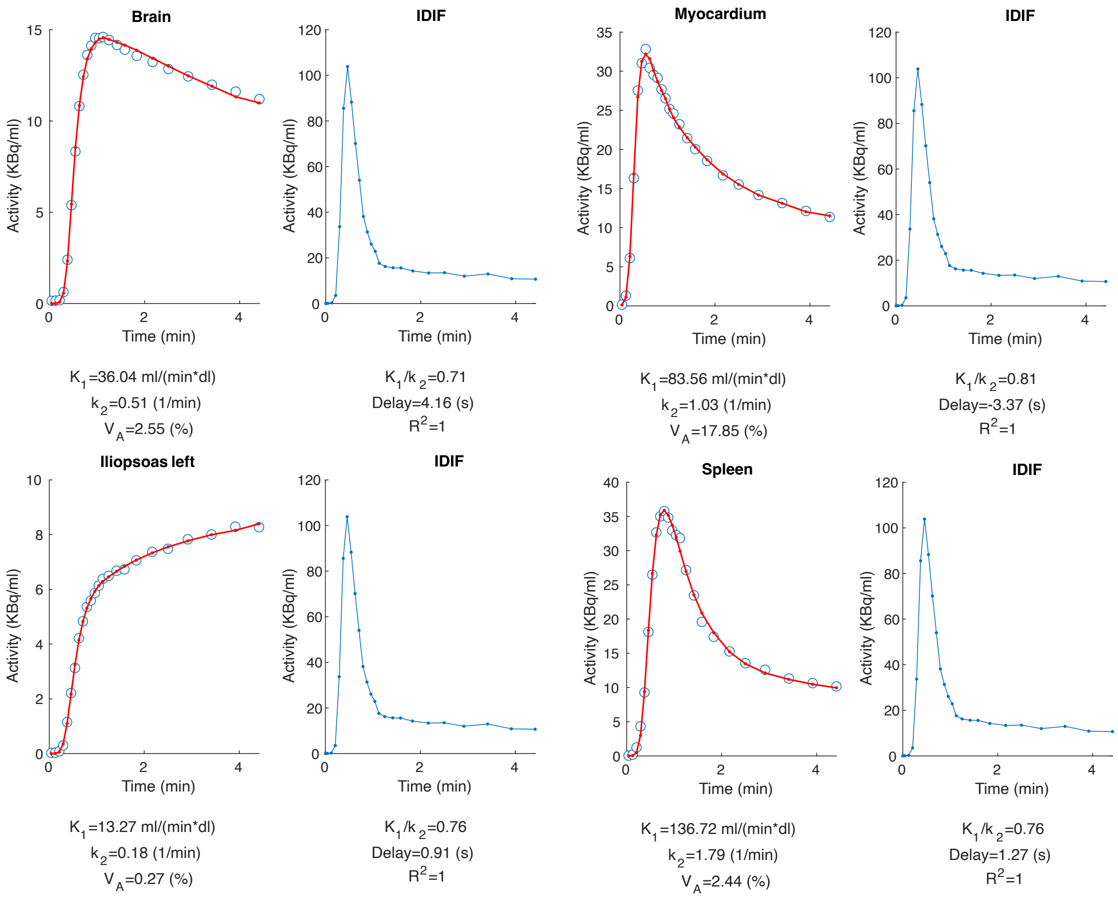

Figure S4. Example model fits

RESULTS

Table S2. Typical TURBO-pipeline processing times time for representative cases of our [^{15}O]H_2O and [^{18}F]FDG data. Average processing times were measured using data from 21 [^{15}O]H_2O subjects and 16 [^{18}F]FDG subjects on our computational server using 25 parallel processes (details below), with CT segmentation done on a GPU. The processing time depends on the number of frames (24 for [^{15}O]H_2O and 34 for [^{18}F]FDG), image matrix size (220 × 220 × 380 for [^{15}O]H_2O and 440 x 440 x 354 for [^{18}F]FDG) and the available computational resources. The TURBO pipeline supports parallelization either by the number of image frames, or by the number of subjects.

Process	[^{15}O]H_2O processing time (mean (sd), minutes)	[^{18}F]FDG processing time (mean (sd), minutes)
CT segmentation	9.1 (0.5)	7.4 (0.9)
CT co-registration	14.8 (0.6)	15.5 (0.6)
PET motion correction	31.4 (2.8)	28.9 (2.0)
TAC extraction	5.6 (0.6)	7.1 (0.4)
Input data processing	1.4 (0.2)	1.7 (0.2)
Quality control	2.0 (0.5)	3.0 (0.5)
ROI-level modelling	0.5 (0.1)	0.3 (0.1)
Voxel-level modelling	9.9 (2.5)*	2.5 (0.2)
Magia brain processing & modelling	16.2 (2.2)	14.5 (1.1)
Total running time	91.0 (7.5)	80.8 (3.6)

System: Intel(R) Xeon(R) Platinum 8468V (97.5M Cache, 2.40 GHz, 900 Gb RAM) with NVIDIA L40 GPU (46 GB VRAM).

* Three-parameter 1TCM NNLS fit; the basis-function method required additional 48 ± 13 (mean ± SD) minutes.

Table S3. Input ROI volumes.

Measure	[^{15}O]H_2O Manual input	[^{15}O]H_2O IDIF	[^{15}O]H_2O p-value*	[^{18}F]FDG Manual input	[^{18}F]FDG IDIF	[^{18}F]FDG p-value*
Volume (ml)	4.0 (2.2)	4.0 (0.7)	0.99	8.2 (2.0)	4.5 (0.9)	p < 0.01
AUC/1000	120.7 (28.2)	120.4 (28.8)	0.53	167.5 (29.2)	167.3 (30.1)	0.84
Dice coefficient	0.5 (0.1)			0.5 (0.1)		

*paired t-test

Table S4. Corrected frame-wise motion (mm) for selected six regions in [^{15}O]H_2O and [^{18}F]FDG test data.

Region	[^{15}O]H_2O		[^{18}F]FDG	
	Mean motion (mm) mean (sd)	Max motion (mm) mean (sd)	Mean motion (mm) mean (sd)	Max motion (mm) mean (sd)
Brain GMctx	0.6 (0.6)	2.1 (1.0)	2.3 (0.9)	6.1 (2.0)
Left iliopsoas	2.4 (0.7)	4.7 (1.2)	3.8 (1.9)	6.9 (3.3)
Liver	5.0 (1.9)	18.7 (7.8)	4.3 (1.9)	18.5 (17.6)
Right kidney	5.1 (1.5)	16.4 (7.5)	4.1 (1.5)	9.9 (3.1)
Spleen	3.1 (0.8)	6.5 (3.3)	3.9 (1.2)	8.0 (2.6
Pancreas	4.4 (1.4)	11.1 (6.1)	4.6 (1.4)	13.5 (6.1)

Table S5. Volumes of manually drawn ROIs and segmented CT ROIs.

Radioligand	Region	Segmented CT volume (ml) mean (sd)	Manual ROI volume (ml) mean (sd)
[^{15}O]H_2O	Liver	1510.7 (464.6)	42.4 (20.6)
	Spleen	181.2 (63.7)	13.9 (5.3)
	Kidney	382.6 (137.0)	47.4 (16.9)
[^{18}F]FDG	Liver	1639.8 (330.5)	29.6 (12.9)
	Iliopsoas	578.9 (92.3)	11.6 (4.5)

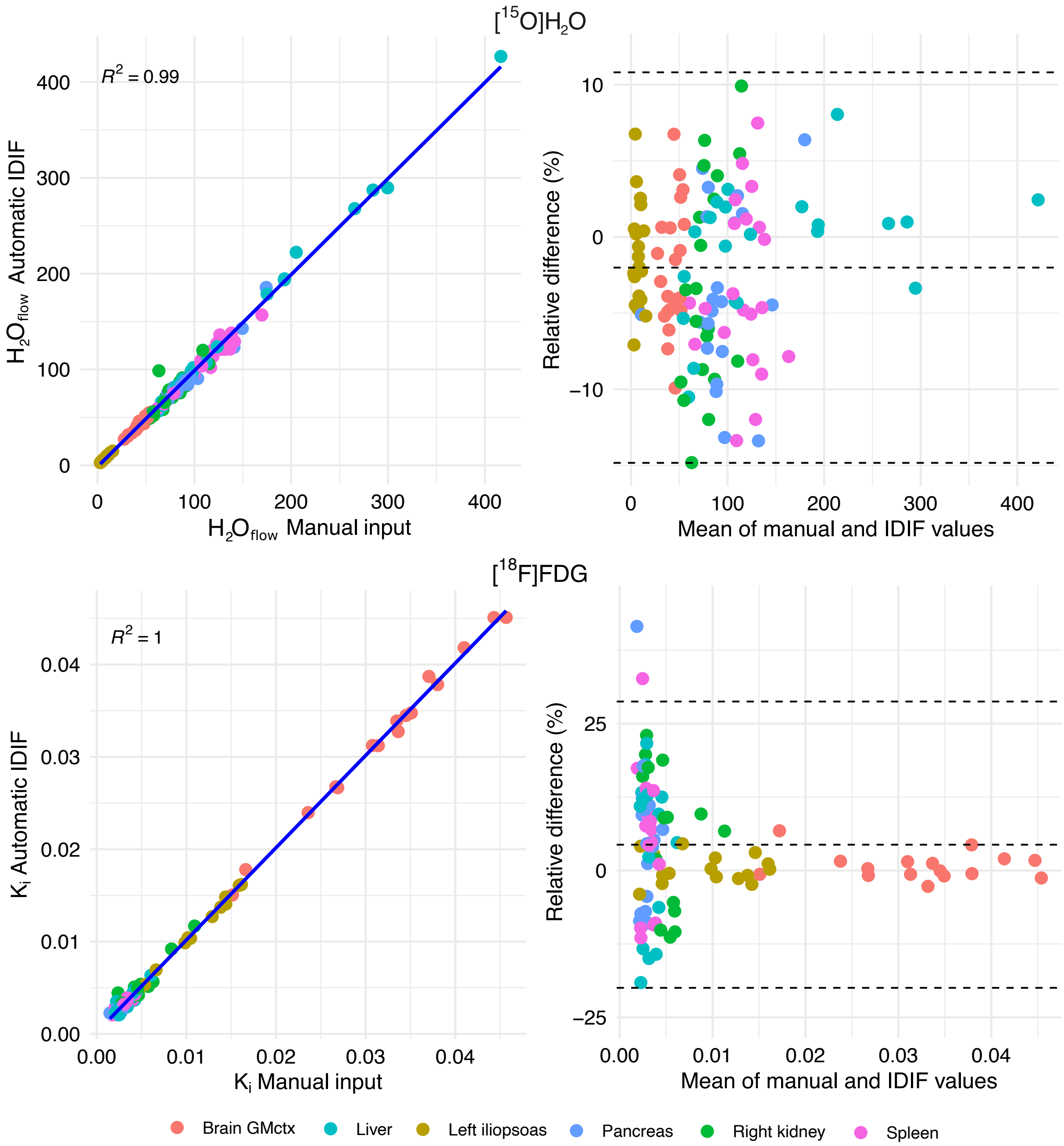

Figure S4. Pearson's correlation and Bland-Altman plots of parameter estimates (H_2O_{flow} = ($K_1(1\text{-}V_A)$ for [^{15}O]H_2O and Patlak K_i for [^{18}F]FDG) in six example regions, calculated using manually derived, and image derived input (IDIF) from descending aorta.

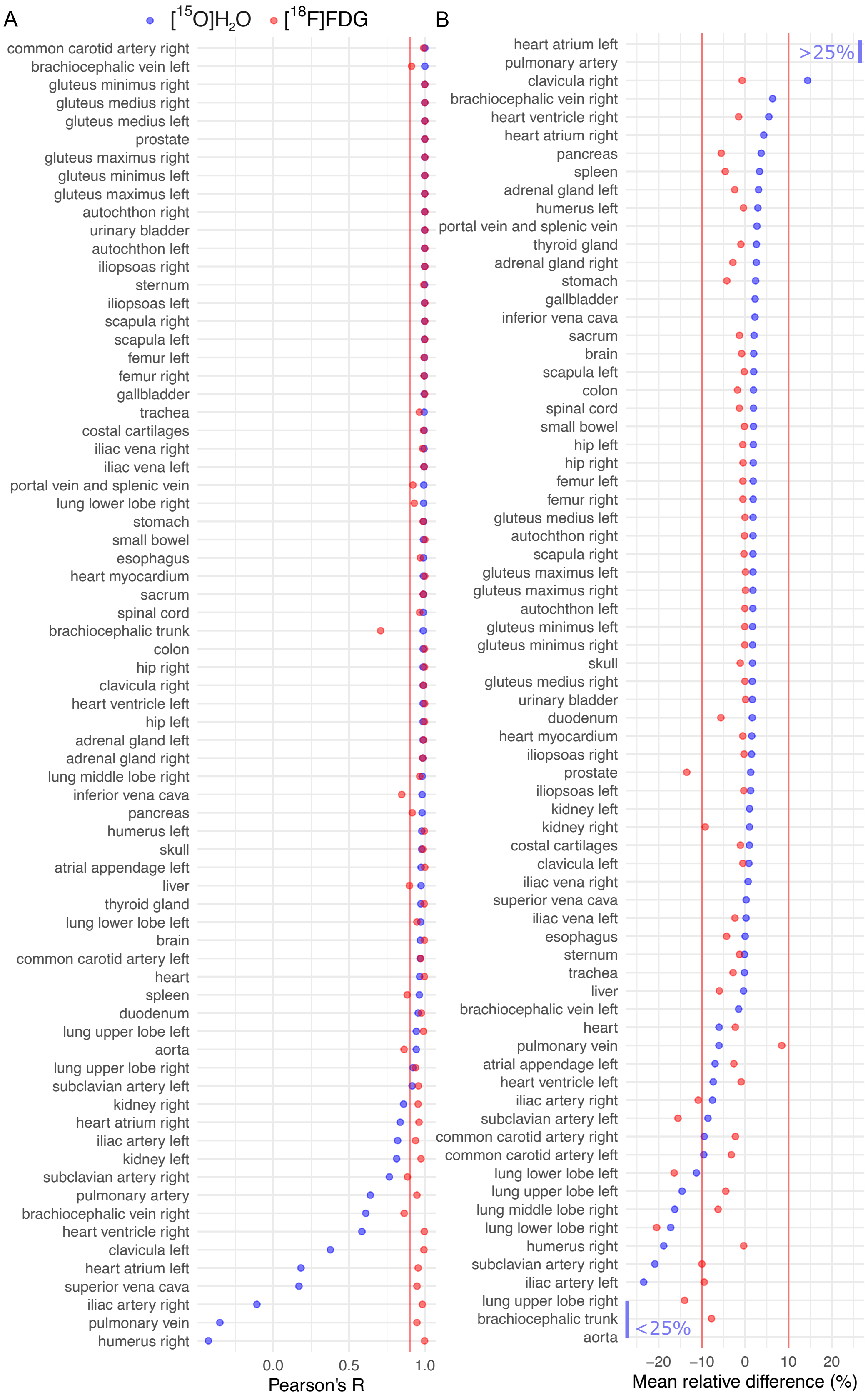

Figure S5. A) Pearson's correlation of model parameter estimates calculated using manually derived, and image derived input (IDIF) from descending aorta. **B)** Mean relative differences between model parameter estimates calculated using manually derived, and image derived input (IDIF) from descending aorta. Regional $H_2O_{flow} = K_1(1\text{-}V_A)$ estimates for $[^{15}O]H_2O$ are coloured in blue and $[^{18}F]FDG$ K_i estimates in red.

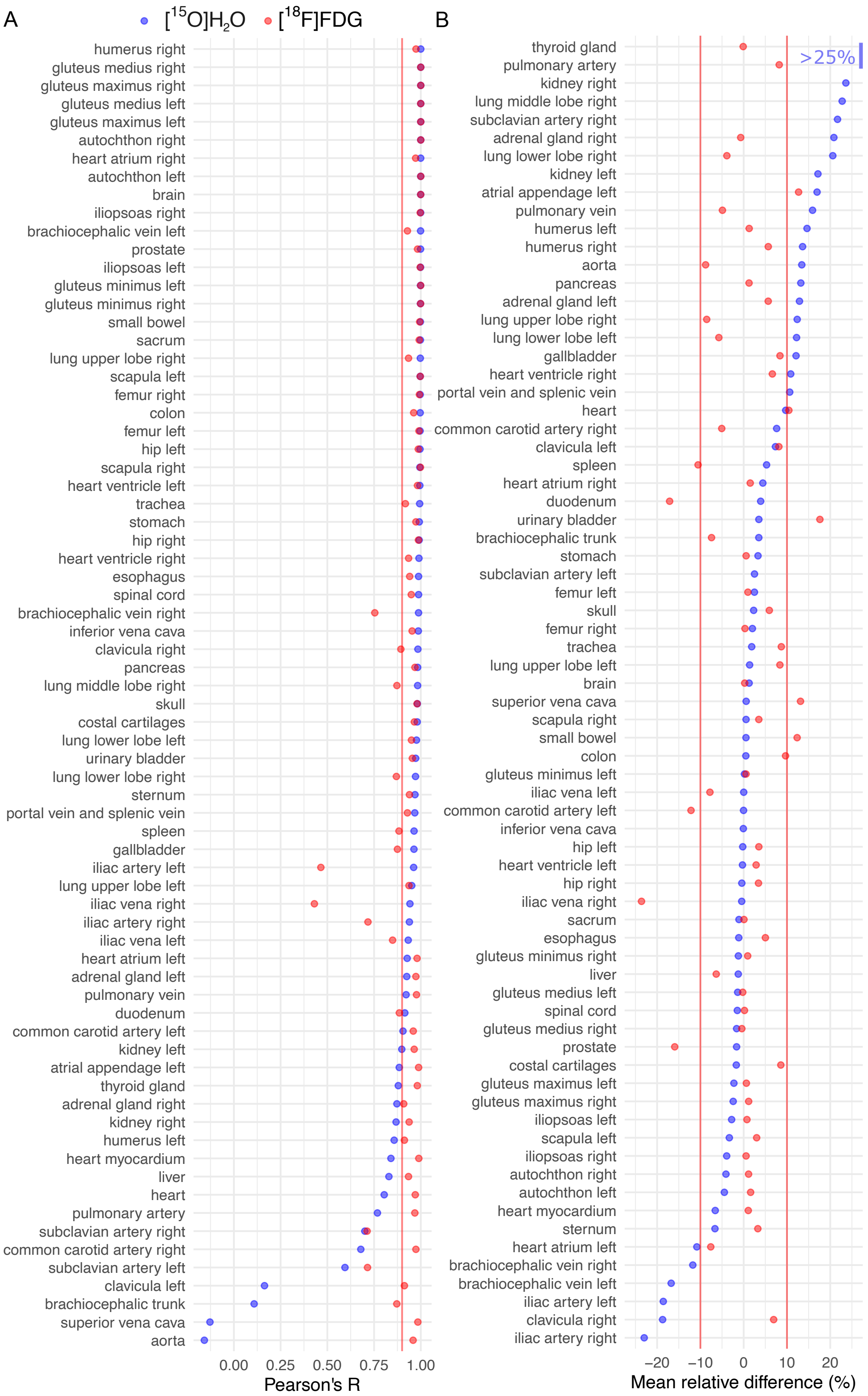

Figure S6. A) Pearson's correlation of model parameter estimates calculated using data with and without motion correction. **B)** Mean relative differences between model parameter estimates calculated using data with and without motion correction. Regional $H_2O_{flow} = K_1(1-V_A)$ estimates for $[^{15}O]H_2O$ are coloured in blue and $[^{18}F]$FDG K_i estimates in red.

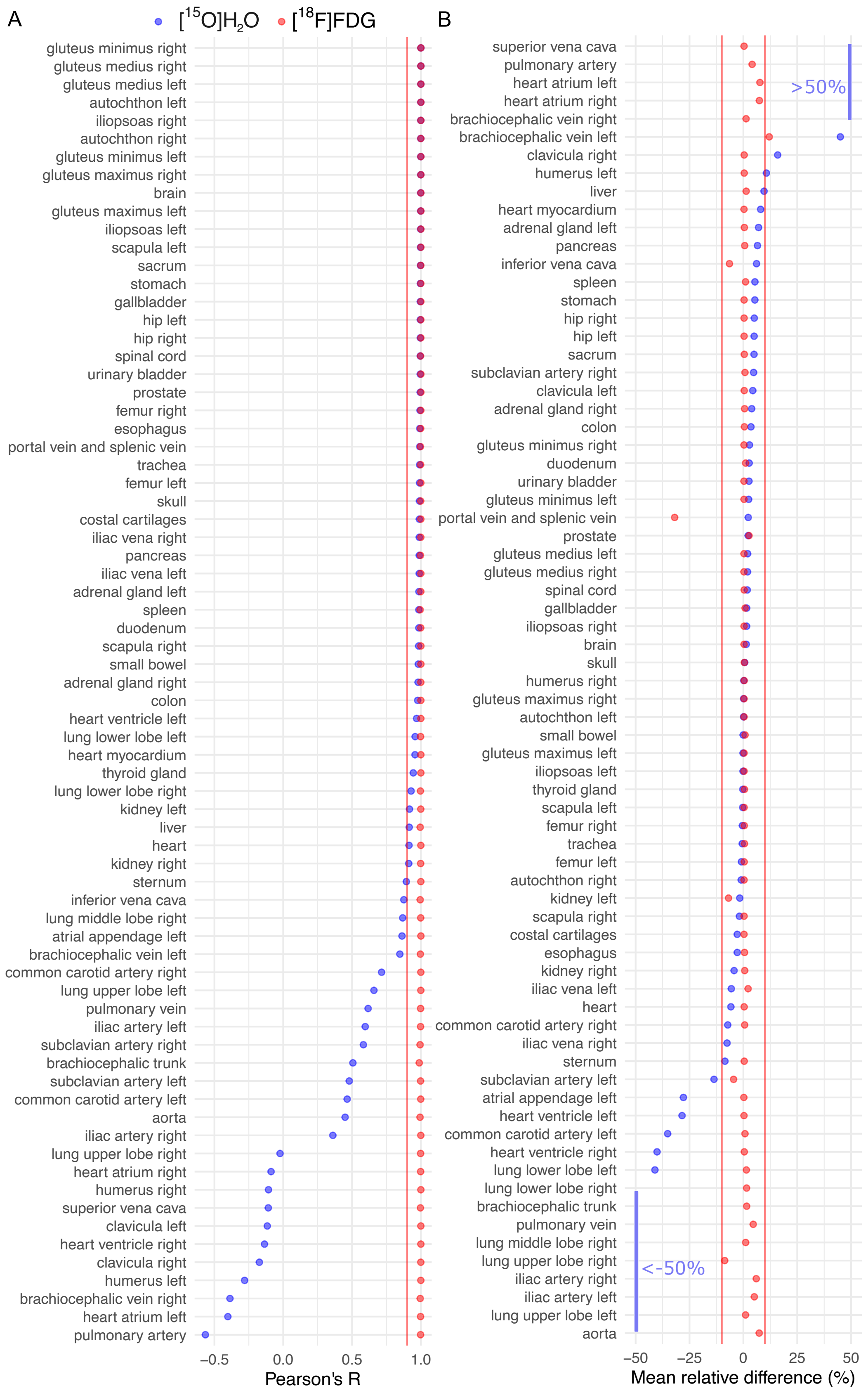

Figure S7. A) Pearson's correlation of model parameter estimates calculated using ROI- and voxel level modelling. Basis function method was used at voxel-level. **B)** Mean relative differences between model parameter estimates calculated using ROI- and voxel level modelling**.** Regional $H_2O_{flow} = K_1(1\text{-}V_A)$ estimates for $[^{15}O]H_2O$ are coloured in blue and $[^{18}F]FDG$ K_i estimates in red.

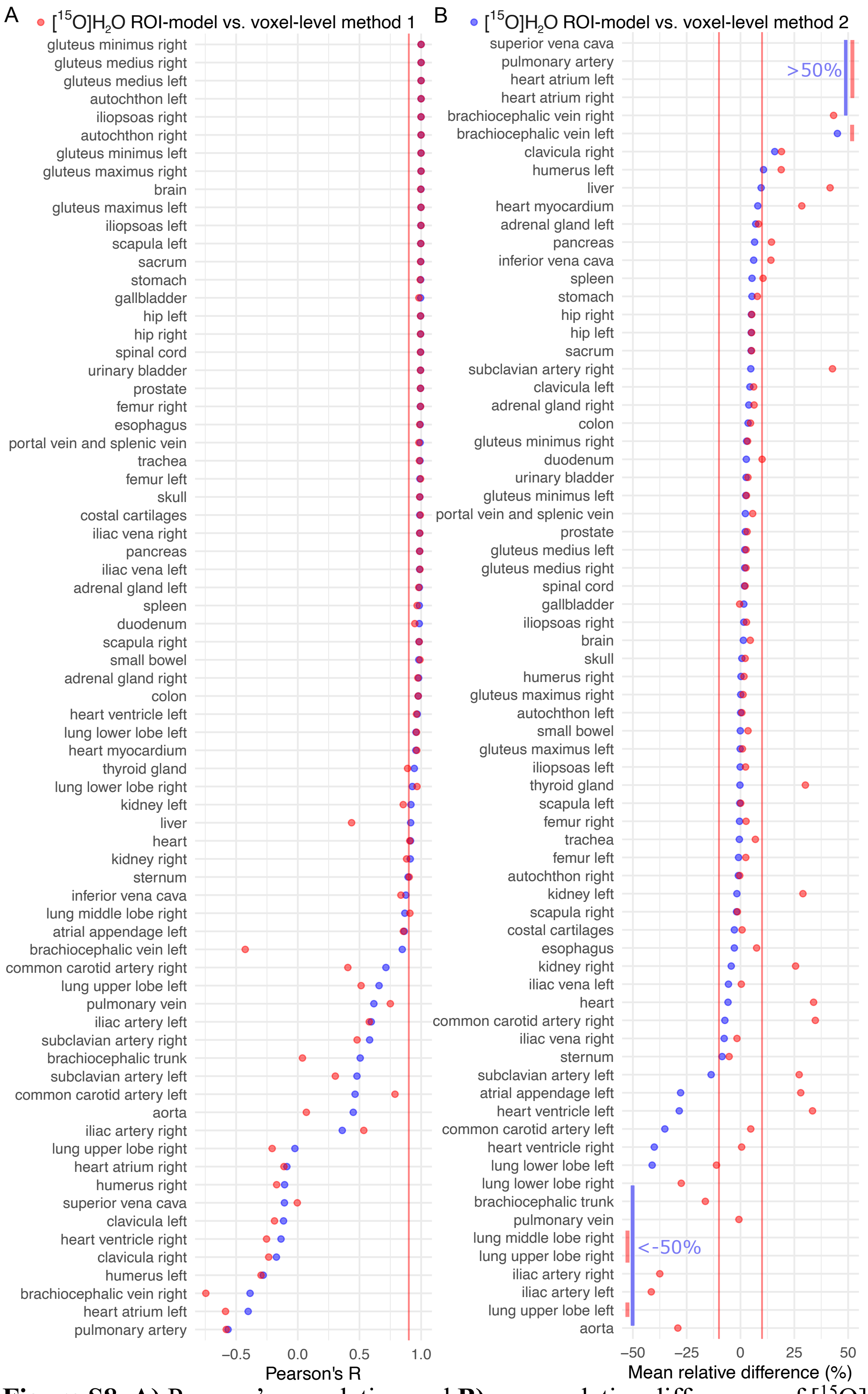

Figure S8. A) Pearson's correlation and **B)** mean relative differences of [15O]H_2O 1-tissue compartment model $H_2O_{flow} = K_1(1\text{-}V_A)$ estimates calculated using ROI- and voxel level modelling. Red markers show the comparison between a three-parameter (K_1, k_2, V_A) ROI-level model and three-parameter voxel-level model (method 1), and blue markers show the comparison between three-parameter ROI level-model and basis function voxel-level model (method 2) with two estimated model parameters (K_1, V_A).

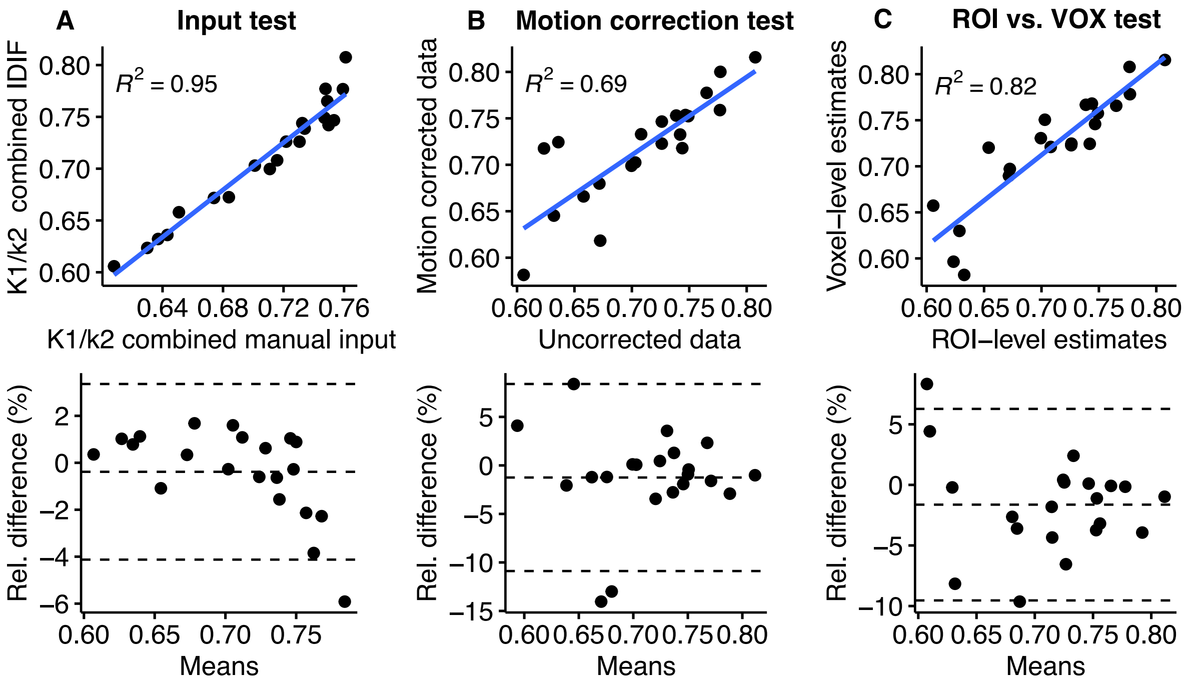

Figure S9. Scatterplots and Bland-Altman plots illustrating correlation and relative difference between $[^{15}O]H_2O$ liver partition coefficient of water ($p=K_1/k_2$) estimates, where **A**) parameter estimates obtained with manual input are compared with parameter estimates calculated using IDIF, **B**) parameter estimates calculated using uncorrected data are compared with parameter estimates calculated using motion corrected data, and **C**) ROI-level parameter estimates are compared with voxel-level parameter estimates.

References:

1. Oikonen V. Multilinear solution for 4-compartment model: I. Tissue compartments in series. 2003. https://doi.org/10.13140/2.1.1598.9762
2. Lawson CL, Hanson RJ. Solving Least Squares Problems. Solving Least Squares Probl. Society for Industrial and Applied Mathematics; 1995.
3. Kudomi N, Slimani L, Järvisalo MJ, Kiss J, Lautamäki R, Naum GA, et al. Non-invasive estimation of hepatic blood perfusion from H215O-PET images using tissue-derived arterial and portal input functions. Eur J Nucl Med Mol Imaging. 2008;35:1899. https://doi.org/10.1007/S00259-008-0796-Z
4. Ziegler SI, Haberkorn U, Byrne H, Tong C, Schosser R, Krieter H, et al. Measurement of liver blood flow using oxygen-15 labelled water and dynamic positron emission tomography: Limitations of model description. Eur J Nucl Med. Springer; 1996;23:169–77. https://doi.org/10.1007/BF01731841
5. DeFronzo RA, Tobin JD, Andres R. Glucose clamp technique: a method for quantifying insulin secretion and resistance. https://doi.org/101152/ajpendo19792373E214. 1979;6.
